# Comparative Evaluation of Ethanolic Extracts Obtained From the Seeds of White and Black Açai Exhibit Antioxidant and Cytoprotective Activity In Vitro

**DOI:** 10.1002/cbdv.71858

**Published:** 2026-10-22

**Authors:** Luana França Calandrini de Azevedo, Estanislaw Tairowite Prestes Correa, Hemilly Casanova Gonçalves Soeiro, Oscar Vinícius Moraes dos Santos, Gustavo Henrique da Silva Lopes, Karina Motta Melo, Vinicius Silva de Carvalho, Andryo Orfi de Almada Vilhena, Jorge das Dores Rissino, Karen Albuquerque Dias da Costa, Alberdan da Silva Santos, Cleusa Yoshiko Nagamachi, Julio Cesar Pieczarka

**Affiliations:** ^1^ Laboratório de Citogenética, Centro de Estudos Avançados da Biodiversidade, Instituto de Ciências Biológicas Universidade Federal do Pará Belém Pará Brazil; ^2^ O Laboratório de Investigação Sistemática em Biotecnologia e Biodiversidade Molecular, Instituto de Ciências Exatas e Naturais Universidade Federal do Pará Belém Pará Brazil; ^3^ CNPQ Researcher Brazil

**Keywords:** antioxidant, cell line, cytoprotective, nutrigenomics, seed

## Abstract

The açai fruit (*Euterpe oleracea*) is valued for bioactive properties beyond nutrition, with its seed typically discarded as waste. This study evaluated ethanolic extracts from black (BA) and white (WA) açai seeds for antioxidant, cytotoxic, cytoprotective, and genotoxic activities in vitro. Using DPPH, MTT, and micronucleus assays on normal (MNP01) and gastric adenocarcinoma (ACP02) cell lines, both extracts showed antioxidant activity (BA ∼30% more effective than WA). Neither cytotoxic nor genotoxic effects were observed, but both exhibited cytoprotective properties in short‐term assays. In clonogenic assays, BA (200 µg/mL) inhibited colony formation in both lines but did not interfere with cisplatin's efficacy. The findings suggest açai seed extracts offer transient cytoprotection, likely due to phenolic content and free radical scavenging, warranting further study for potential therapeutic applications.

## Graphical abstract

Açai seed extracts (black/white) showed antioxidant activity (black ∼30% stronger), no cytotoxicity or genotoxicity, and transient cytoprotection in normal and cancer cell lines. Black extract (200 µg/mL) inhibited colony formation without reducing cisplatin efficacy. Phenolic‐rich seed waste warrants further therapeutic study.

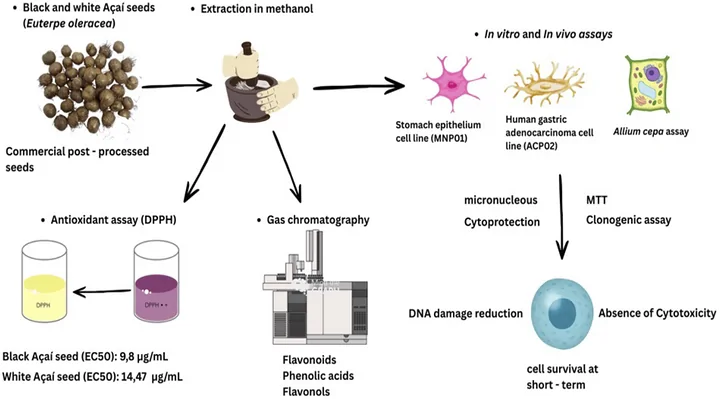

## 1 Introduction

*Euterpe oleracea* (açai palm) is recognized as the most productive palm species in the Amazon region, primarily due to the comprehensive economic utility of the entire tree. Throughout the years, this species has been harvested by riverside communities in the Amazon, who utilize açai palm leaf fibers for crafting hats, mats, bags, and baskets. Furthermore, the palm heart—highly valued and traded both nationally and internationally—is extracted from its trunk [1, 2]. Notably, the açai palm's most significant asset is its fruit, the açai berry, which has attracted considerable attention owing to increasing research substantiating its health benefits for humans. The fruit is incorporated into various products, such as ice cream, yogurt, creams, and liqueurs, in forms ranging from frozen pulp and powder to fresh consumption [3, 4, 5].

Phenotypic variation is observed in the coloration of ripe açai fruit. The most widely recognized variant is black açai, which exhibits a purple or violet hue following processing, while white açai has fruits with an opaque green color, resulting in a creamy greenish beverage [6]. The fruit pulp constitutes 5% to 15% of the total fruit volume, depending on type and ripeness, with the remainder primarily composed of the seed, a byproduct that is typically discarded after juice extraction [7]. This seed has garnered research interest across multiple fields due to concerns regarding disposal and potential reuse, as daily açai production generates substantial amounts of organic waste. Additionally, numerous studies have documented antioxidant, vasodilator, antihypertensive, emphysema‐protective, and antimicrobial properties associated with açai [8, 9, 10, 11, 12, 13].

The presence of phenolic compounds, including vanillic, caffeic, and syringic acids, as well as anthocyanins, a particular class of flavonoids responsible for the deep purple coloration of black açai pulp, contributes to the antioxidant properties commonly associated with the fruit [14, 15, 16]. However, anthocyanins are primarily associated with the pulp and were not identified in the ethanolic seed extracts analyzed in the present study. Therefore, the phytochemical composition of the pulp should be distinguished from that of the seeds when discussing the bioactive compounds of açai. These compounds inhibit or mitigate the effects of free radicals within the body. Açai is increasingly recognized as a functional food, providing benefits beyond basic nutrition, and is frequently studied in the context of nutrigenomics [17, 18]. Nutrigenomics assesses how specific dietary components can modulate gene expression, with antioxidant activity being particularly notable for its role in combatting various diseases, including neoplasms [19]. By 2030, it is projected that there will be 21.4 million new cancer cases globally; however, this figure may be influenced by modifications in dietary practices, such as increased intake of exogenous antioxidants or regular consumption of plant‐derived compounds capable of scavenging free radicals [20, 21, 22].

Free radicals exhibit significant chemical reactivity due to their possession of an odd number of electrons, which predisposes them to seek stabilization by acquiring an additional electron [23]. These molecules are formed naturally within the body, as oxidation is essential to aerobic organisms; under normal conditions, free radicals are eliminated by endogenous antioxidant mechanisms [24]. Nevertheless, exposure to various external factors may disrupt the balance between free radical generation and removal, overwhelming the antioxidant defense system and resulting in oxidative stress. In such circumstances, the dietary inclusion of exogenous antioxidants, such as açai, plays a crucial role in maintaining oxidative equilibrium and supporting overall health [25, 26].

To bridge the gap between initial hazard identification and mammalian relevance, this study employs a tiered in vitro testing strategy. The first tier utilizes the standardized *Allium cepa* bioassay, a model recommended by the United Nations Environmental Programme (UNEP) and the Organization for Economic Co‐operation and Development (OECD) for ecotoxicological screening due to its correlation with other test systems [27]. This assay is widely acknowledged for its rapidity, low cost, and robust cytogenetic endpoints—such as mitotic index, chromosomal aberrations, and micronuclei formation—which are comparable to those observed in mammalian systems due to the evolutionary conservation of cell cycle checkpoints and DNA repair mechanisms [28]. The *A. cepa* test serves as a reliable sentinel system, allowing for preliminary cytotoxicity threshold determination while adhering to the 3Rs (Replacement, Reduction, and Refinement) principles of animal testing [29]. Its use is not a substitute for human‐based models, but rather a strategic, ethical, and economical preliminary filter that guides subsequent, more resource‐intensive mammalian cell culture experiments.

The purpose of this study is to assess the antioxidant properties of ethanolic extracts from both white and black açai seeds. It also examines how well these extracts protect cell lines before exposure to doxorubicin, while exploring which concentrations might cause cytotoxic or genotoxic effects. The goal is to identify an antioxidant, cytoprotective, and safe concentration under the conditions tested.

## 2 Materials and Methods

### 2.1 Sample Collection and Preparation

Ethanolic extracts of açai were produced at the Laboratory for Systematic Research in Biotechnology and Fine Chemistry (LabISisBio). Black and white açai seeds were collected as agro‐industrial waste in the municipality of Santa Barbara, Pará (latitude 1°15',05.13''S, longitude 48°16',35.96'W) in 06/02/2018. Dr. Maria do Socorro Padilha de Oliveira identified the material. The voucher numbers are IAN 183.053 (black açai) and IAN 184.142 (with açai**)**. The material is deposited in the IAN Herbarium (located in Belém, Pará, Brazil, at Embrapa Amazônia Oriental). The material was dried for 3 days in air‐circulating ovens at 60°C and then ground in a hammer mill with a 1 mm sieve. The metabolites present in 1 kg of *E. oleracea* seeds (black and white) were extracted at a temperature of 35°C with 1.5 L and at 45°C with 3.0 L of ethanol. Each extraction was carried out for 30 min under ultrasound‐assisted agitation (UNIQUE ultrasonic bath—USC – 1800, 40 KHz frequency, 155 Watts power), resulting in the ethanolic extracts of black açai (BA) and white açai (WA).

### 2.2 Chemical Characterization of the Samples

#### 2.2.1 Identification of Tannins by Wet Method

In a 10 mL test tube, 50 mg of BA and WA were added, along with 2.0 mL of distilled water, and the mixture was shaken until completely dissolved. Then, three drops of Ferric Chloride (FeCl_3_) were added. At 10.0%, a color change was observed, where the presence of green coloration indicated the presence of condensed tannins and the presence of blue coloration indicated the presence of hydrolyzable tannins.

#### 2.2.2 Thin‐Layer Chromatography (TLC)

The extracts (BA and WA) were applied to silica gel plates (3 cm wide, 5 cm long, and 4 cm chromatographic path). Elution was performed using the system Ethyl Acetate: Acetic Acid: Formic Acid: H_2_O (10:1:1:2.6). The metabolites investigated and their respective revealing agents were condensed tannins (vanillin 1.0% and sulfuric acid 10.0%, both in ethanol) (VAS+H_2_SO_4_), fatty acids (phosphomolybdic acid) (PMA), and phenolic compounds (Ferric Chloride, 10.0% in ethanol) (FeCl_3_), anthraquinones, anthrones, anthronols, coumarins (Potassium Hydroxide, 1.0% in ethanol and ultraviolet radiation at a wavelength of 365 nm) (KOH+UV), alkaloid (Dragendoff), flavonoids (diphenylboryloxyethylamine, 1.0% in methanol and polyethylene glycol 5.0%) (NP+PEG).

#### 2.2.3 Extraction, Hydrolysis, and Derivatization of Phenolic Compounds

##### 2.2.3.1 Extraction

The phenolic compounds present in 1000 g of *E. oleracea* Mart. seeds from black and white açai samples (chemotypes 1 and 2, respectively) were extracted for 30 min at 45°C using 3.0 L of analytical‐grade ethanol under ultrasound‐assisted agitation. The extraction was performed in an ultrasonic bath (UNIQUE USC‐1800, 40 kHz frequency, 155 W power) [30].

##### 2.2.3.2 Hydrolysis and Extraction of Phenolic Compounds

The ethanolic seed extract was subjected to acid hydrolysis by adding 500 µL of 6 mol L hydrochloric acid (HCl), followed by vortex agitation for 2 min and adjustment of the solution to pH 2. The system was then subjected to ultrasound‐assisted agitation for 30 min at 60°C.

The phenolic compounds were extracted three consecutive times using 500 µL of ethyl acetate (EtOAc) in each extraction. After each extraction, the samples were centrifuged at 10 000 rpm for 2 min. The supernatants were collected and evaporated to dryness under a nitrogen atmosphere. All procedures were performed under inert conditions.

##### 2.2.3.3 Derivatization of the Phenolic Concentrate

The dried phenolic concentrate was derivatized by adding 200 µL of *N,O*‐bis(trimethylsilyl)trifluoroacetamide containing 1% trimethylchlorosilane (BSTFA + 1% TMCS). The mixture was vortexed for 2 min and subsequently subjected to ultrasound‐assisted agitation for 30 min at 60°C. After derivatization, 500 µL of ethyl acetate was added, followed by vortex agitation for 2 min and centrifugation at 10 000 rpm for 2 min. The supernatant was transferred to a capped glass vial fitted with a septum and subsequently analyzed by GC–MS.

#### 2.2.4 Chromatographic Analysis by GC–MS

Gas chromatography–mass spectrometry (GC–MS) analyses were performed using a Thermo Scientific Trace 1300 gas chromatograph coupled to a Thermo Scientific ISQ single‐quadrupole mass spectrometer and equipped with an AI 1310 autosampler. Chromatographic separation was performed using a ZB‐5HT Inferno capillary column (30 m × 0.25 mm i.d. × 0.10 µm film thickness), with helium as the carrier gas at a flow rate of 1.0 mL min.

The derivatized samples were injected at a volume of 1.0 µL in splitless mode. The injector temperature was set at 250°C. The oven temperature program started at 200°C, which was maintained for 2 min, followed by an increase to 300°C at a rate of 20°C min, with a final hold of 10 min. The mass spectrometer interface and ion source temperatures were set at 275°C and 230°C, respectively. A solvent delay of 5 min was used, and the mass range was set from *m/z* 50 to 1000. Electron ionization was performed at 70 eV.

Compound identification was based on comparisons between the acquired mass spectra and those available in the NIST2011, Wiley2009, and FAMES2011 commercial spectral libraries. Thus, derivatization was performed before GC–MS analysis to improve the volatility and chromatographic behavior of the phenolic compounds.

### 2.3 DPPH (2,2‐Diphenyl‐1‐picrylhidazyl) Antioxidant Activity Test

The determination of antioxidant activity was performed in triplicate using a spectrophotometric method. DPPH was diluted in methanol at a concentration of 0.04 mg/mL. For the reaction, 900 µL of the DPPH solution and 100 µL of the extracts diluted in methanol at concentrations of 6.25–400 µg/mL were used. The mixture reacted for 20 min in a light‐protected environment, and after this, the absorbances of the samples at different concentrations were measured using a spectrophotometer (Epoch Biotek) with the Gen5 (2003) version 2.03.1 program.

The results were expressed as a percentage of DPPH radical inhibition (% inhibition), using absorbances for the calculation through the formula: % Inhibition = {[A1 − A2 + A3] / A1} × 100, where A1: absorbance of DPPH, A2: absorbance of DPPH + tested and diluted extract, and A3: absorbance of methanol + tested and diluted extract. After that, the extract concentration necessary to capture 50% of the DPPH free radical (EC_50_) was also calculated using the Graphpad program Prism version 6.01 [31].

### 2.4 Cell Line and Culture Conditions

For all tests, two gastric cell lines were used: one non‐neoplastic MNP01 and another gastric adenocarcinoma ACP02 (RRID:CVCL_IK51), both kindly provided by the Human Cytogenetics and Toxicological Genetics Laboratory of the Institute of Biological Sciences at the Federal University of Pará. These were cultured in DMEM culture medium supplemented with 10% fetal bovine serum (FBS).

### 2.5 Experimental Design of In Vitro Assays

In this research, four biological assays were performed: the MTT assay (3‐[4,5‐dimethylthiazol‐2‐ yl] ‐2,5‐diphenyltetrazolium bromide), with seeding of cells in 96‐well plates; the micronucleus test, in which this step was performed in 12‐well plates; the clonogenic assay, performed in 12‐well plates; and the *A. cepa* test, conducted in Petri dishes.

In the MTT and micronucleus tests, for 48‐h exposure, concentrations of 6.25–400 µg/mL of BA and WA were used. DMEM culture medium was used as a negative control (NC), and since DMSO was used as a dilution vehicle (0.1%), it was also tested, diluted in culture medium, at the same concentration. The positive control (PC) for MTT was performed using doxorubicin (DOX) (200 µg/mL), and for the micronucleus, colchicine (0.02 µg/mL) was used, according to literature [32]. In this last assay, 24 h before cell removal, cytochalasin B (6 µg/mL) was added to each well.

#### 2.5.1 Evaluation of MTT Cytotoxicity and Cytoprotection

For cytoprotective assessment, using the MTT assay, exposure was performed as described in the previous item, but concomitantly with DOX diluted in DMEM culture medium (200 µg/mL) in all treatments.

Following the seeding and exposure steps, the cells in the wells were subjected to 100 µL of MTT (5 mg/mL) for 3 h. After removing the MTT, 100 µL of DMSO was added to each well for 1 h. After this, the absorbance was measured using a spectrophotometer (Epoch Biotek) with the Gen5 (2003) version 2.03.1 program. The absorbance was converted to survival (%S) using the formula: %S = 100 × [(Atested—Blank) / (Acontrol—Blank)], where “A” is the absorbance of each well; the well without medium and cells was considered the blank [33].

#### 2.5.2 In Vitro Micronucleus Test

Following the steps already described, the cell lines were trypsinized and transferred to a Falcon tube to which hypotonic solution (KCl) was added, followed by Carnoy's fixative with methanol and acetic acid (3:1). After centrifugation and removal of the supernatant, 1 mL of Carnoy's fixative was added, and the tube was stored for later analysis. The evaluation was performed in a blinded test, with 500 total cells analyzed and quantified to determine the Cytokinesis Blocking Proliferation Index (CBPI) using the formula: CBPI = [M1+2(M2)+3 (M3)+4(M4)]/N; where M1 to M4 are the number of cells with 1, 2, 3, and 4 nuclei, respectively, and N is the total number of viable cells. The counting of binucleated cells continued until 1000 cells were counted for micronucleus observation and determination of their frequency using the formula fMN = number of MN / 1000 [34].

### 2.6 *Allium cepa* Cytoprotection Test

Onion seeds (*A. cepa*) were germinated on filter paper moistened with distilled water for 5 days. For the antimutagenic evaluation, the seeds were exposed to different concentrations of ACE (25–200 µg/mL) in pre‐treatment, simultaneous treatment, and post‐treatment protocols with paracetamol (0.3 mg/mL) as a mutagenic agent. After treatment, the root tips were fixed in Carnoy's solution, hydrolyzed in 1N HCl, stained with 2% acetic orcein, and analyzed microscopically. A total of 4000 cells per treatment were evaluated for micronucleus frequency, chromosomal aberrations, and mitotic index.

### 2.7 Clonogenic Assay

For colony formation assessment, 150 cells per well were seeded in 12‐well plates and allowed to adhere for 24 h before treatment with EEAS (25–200 µg/mL) and cisplatin (20 µM) for 24 h. After treatment, the cells were kept in fresh medium for 14–18 days (depending on the cell line), fixed, stained with Giemsa, and the colonies (≥50 cells) were manually counted.

### 2.8 Statistical Analysis

Data normality was assessed using the Kolmogorov‐Smirnov test. For datasets with normal distribution (*p* > 0.05), differences between groups were analyzed using one‐way ANOVA followed by Tukey's *post‐hoc* test. For datasets that deviated from normality (*p* ≤ 0.05), the non‐parametric Kruskal‐Wallis test was used, followed by the Student‐Newman‐Keuls multiple comparison test [35]. All analyses were performed using the Bioestat 5.0 program, with *p* ≤ 0.05 considered statistically significant.

## 3 Results

### 3.1 Chemical Characterization of the Samples

The presence of fatty acids, phenols, and condensed tannins was observed in both extracts analyzed. The compounds found and their respective quantities are presented in Table 1.

**TABLE 1:** Chemical profile of ethanolic extracts of *Euterpe oleracea* seeds obtained by chromatographic analysis.

Compounds	BA (%)	WA (%)	TR	FIT
Pyrogallol	—	0.35	10.10	872
Para‐hydroxybenzoic acid	—	1.57	10.36	834
Benzoic acid	2.47	—	10,41	816
Phloroglucinol	24.71	24.78	10,73	894
4‐Hydroxymandelic acid	0.84	0.99	11,32	679
Vanillic acid	0.06	0.00	12,20	725
Azelaic acid	0.00	0.00	12,42	818
3,4‐Dihydroxybenzoic acid	15.12	15.26	12,82	892
Myristic acid	9.35	7.49	12,89	924
4‐Hydroxyphenyllactic acid	1.49	1.21	13,58	876
3,4‐Dihydroxymandelic acid	0.26	1.62	13,91	823
Palmitic acid	12,11	12.06	14,82	905
Catecholactate	18.79	18.32	15,20	930
2,6‐Dihydroxybenzoic acid	0.00	0.31	15,77	792
trans‐9‐Octadecenoic acid	8.39	9.54	16,30	931
Stearic acid	6.42	6.31	16,56	857
Derivative of catechin	0.05	0.19	19,13	760
NI	—	0.55	18.08	
NI	2.04	—	19.13	
NI	—	1.08	19,92	
NI	—	0.37	20.02	
NI	2.17	1.6	20.76	
NI	2.04	—	21.19	
NI	0.43	0.51	23.60	
NI	1.16	—	24.07	

*Note*: Values are expressed as a percentage (%) of the peak area of the chromatogram.

Abbreviations: BA, Black açai; WA, White açai; FIT, spectral correspondence with reference; NI, not identified; RT, retention time.

The compounds presented in Table 1 correspond to the constituents identified in the derivatized ethanolic extracts of açai seeds by GC–MS. Although anthocyanins are important bioactive compounds in black açai pulp, they were not identified in the seed extracts under the analytical conditions used in this study. Consequently, anthocyanins are not included in Table 1. The chromatograms can be found at supplementary Figures S1 for white açai and S2 for black açai.

### 3.2 Evaluation of Antioxidant Activity

The antioxidant activity observed from the DPPH free radical scavenging demonstrates high potential, in a dose‐dependent manner for both extracts; however, black açai showed greater antioxidant activity, with an EC_50_ of 9.8 µg/mL (95% CI: 6.8–12.55 µg/mL), while that of white açai showed an EC_50_ of 14.47 µg/mL (95% CI: 7.05–14.59 µg/mL) µg/mL (Figure 1). Data extracted from the dose‐response curve shown in Figure 1 are presented as a table in the supplementary tables: Table S1 for black açai and Table S2 for white açai.

**FIGURE 1:**
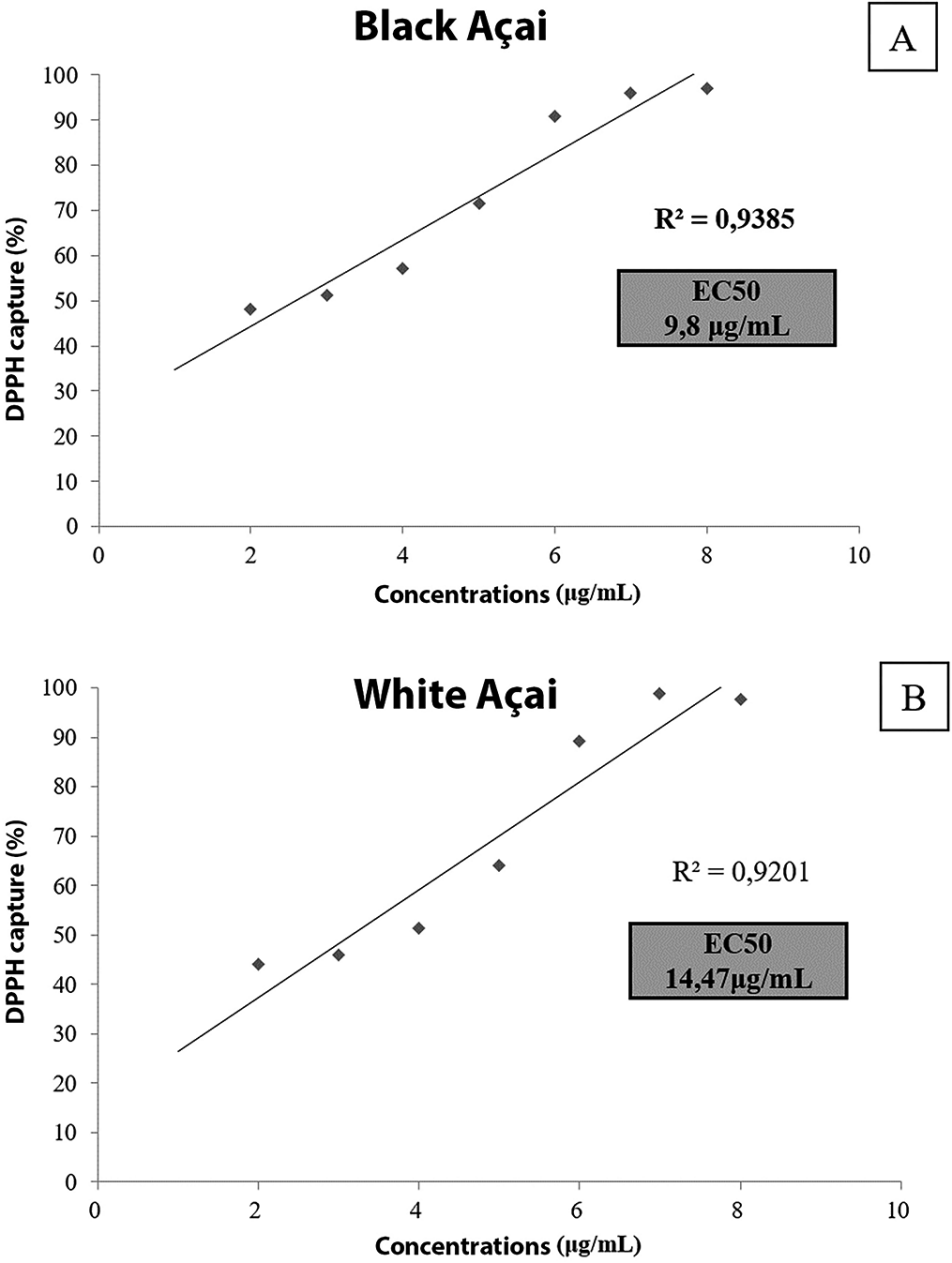
Correlation between DPPH free radical scavenging and the concentrations used. (A) Black açai extract and its respective EC_50;_ (B) White açai extract and its respective EC_50_. Linear regression test.

### 3.3 Assessment of Cytotoxic Activity

After 48 h of exposure in the MTT test, results showed that black and white açaí extracts did not display toxicity in the gastric adenocarcinoma cell line (ACP02). In contrast, the normal gastric cell line (MNP01) experienced reduced viability at the highest tested concentration (400 µg/mL) of both extracts (Figure 2).

**FIGURE 2:**
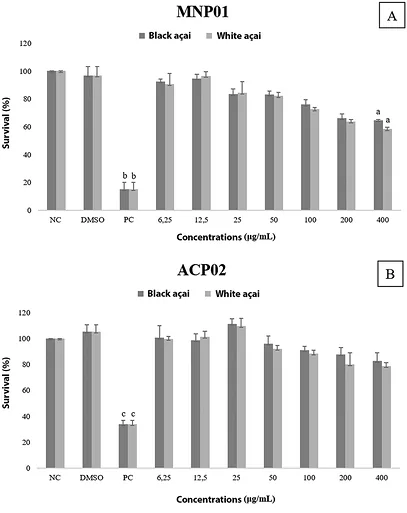
Percentage of mean cell survival with their respective standard deviations, exposed for 48 h to extracts of black and white açai seeds, concomitant with exposure to doxorubicin. (A) Normal gastric cells (MNP01); (B) Gastric adenocarcinoma cells (ACP02). Test: Tukey‐ANOVA, (*p* < 0.05). NC: negative control, PC: positive control. ^a^Differs from NC and DMSO; ^b^differs from all treatments; ^c^differs from 50, 25, 12.5, 6.25 µg/mL, NC, and DMSO.

### 3.4 Evaluation of Cytoprotective Activity

Cytoprotective activity was demonstrated in both ACP02 and MNP01 cell lines, with white and black açai seed extracts at concentrations of 200 and 400 µg/mL providing protection against doxorubicin‐induced damage after 48 h of treatment (Figure 3).

**FIGURE 3:**
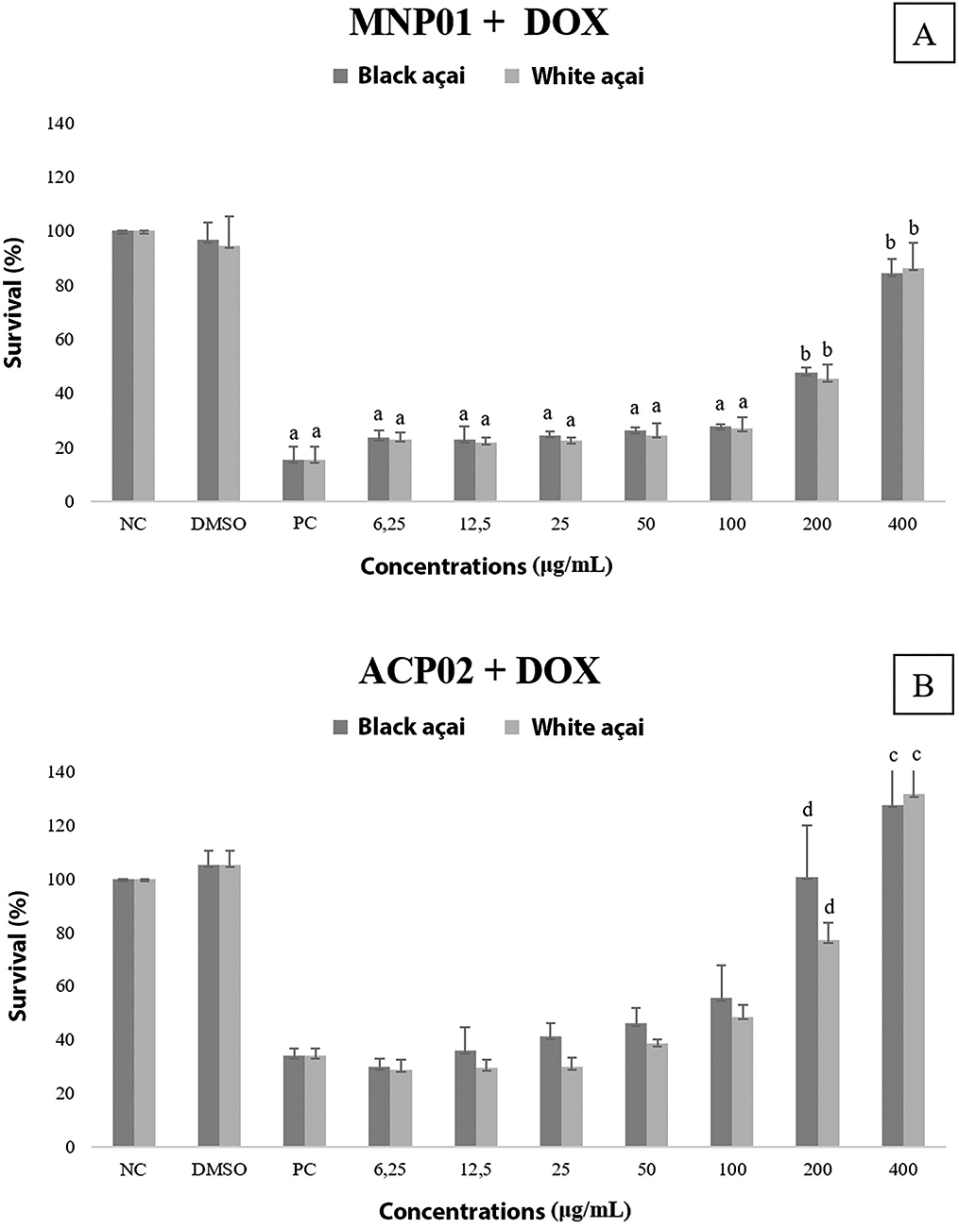
Percentage of mean cell survival with their respective standard deviations, exposed for 48 h to extracts of black and white açai seeds, concomitant with exposure to doxorubicin. (A) Normal gastric cells (MNP01); (B) Gastric adenocarcinoma cells (ACP02). Test: Tukey‐ANOVA, (*p* < 0.05). NC: negative control, PC: positive control and DOX: doxorubicin. ^a^Differs from NC and DMSO; ^b^differs from all treatments; ^c^differs from all treatments except NC and DMSO; ^d^differs from PC, 6.25 and 12.5 µg/mL.

### 3.5 Evaluation of Genotoxic Activity

The micronucleus test showed that MNP01 did not cause micronuclei formation or changes in the Cytokinesis‐block proliferation index (CBPI) (Table 2).

**TABLE 2:** Frequency of micronuclei (fMN) and Cytokinesis‐block proliferation index (CBPI) of non‐neoplastic gastric cells (MNP01) exposed to ethanolic extract of black açai and white açai.

MNP01 cell line		
Black açai		
Treatment	**fMN**	**CBPI**
NC	0.009(±0.006)^a^	1.47 (±0.03)
DMSO (0.1%)	0.003(±0.001)^a^	1.42 (±0.001)
25 µg/mL	0.006(±0.001)^a^	1.39 (±0.03)
50 µg/mL	0.007(±0.001)^a^	1.45 (±0.005)
100 µg/mL	0.005(±0.002)^a^	1.2(±0.16)
200 µg/mL	0.068(±0.06)^a^	1.43 (±0.01)
PC	0.04(±0.009)	1.37 (±0.02)

White Açai		
Treatment	**fMN**	**CBPI**
NC	0.009(±0.006)^a^	1.47 (±0.03)
DMSO (0.1%)	0.003(±0.001)^a^	1.42 (±0.09)
25 µg/mL	0.005(±0.001)^a^	1.42 (±0.03)
50 µg/mL	0.006(±0.002)^a^	1.39 (±0.009)
100 µg/mL	0.005(±0.002)^a^	1.46 (±0.03)
200 µg/mL	0.005(±0.00)^a^	1.39 (±0.01)
PC	0.07(±0.001)	1.37 (±0.01)

Abbreviations: NC, negative control; PC, positive control.

^a^ Differs from PC.

In the gastric adenocarcinoma cell line (ACP02), only the two highest concentrations (100 and 200 µg/mL) of BA treatment showed no significant difference compared to the positive control. Additionally, there was no observed variation in the CBPI (Table 3).

**TABLE 3:** Frequency of micronuclei (fMN) and proliferation cytokinesis blockade index (PCBI) of gastric adenocarcinoma cells (ACP02) exposed to ethanolic extract of black açai and white açai.

ACP02 cell line		
Black açai		
Treatment	**fMN**	**CBPI**
NC	0.01 (±0.006)^a^	1.33 (±0.12)
DMSO (0.1%)	0.00 (±0.001)^a^	1.46 (±0.04)
25 µg/mL	0.01 (±0.001)^a^	1.36 (±0.01)
50 µg/mL	0.01 (±0.001)^a^	1.40 (±0.04)
100 µg/mL	0.01 (±0.004)	1.38 (±0.03)
200 µg/mL	0.02 (±0.009)	1.47 (±0.03)
PC	0.025 (±0.007)	1.34 (±0.09)

White açai		
Treatment	**fMN**	**CBPI**
NC	0.011 (±0.006)^a^	1.33 (±0.12)
DMSO (0.1%)	0.005 (±0.002)^a^	1.46 (±0.04)
25 µg/mL	0.011 (±0.002)^a^	1.41 (±0.03)
50 µg/mL	0.006 (±0.001)^a^	1.45 (±0.46)
100 µg/mL	0.001(±0.001)^a^	1.39 (±0.06)
200 µg/mL	0.008 (±0.001)^a^	1.33 (±0.08)
PC	0.025 (±0.007)	1.34 (±0.09)

Abbreviations: NC, negative control; PC, positive control.

^a^ Differs from PC.

### 3.6 *Allium cepa* Test

The results showed a statistically significant decrease in the micronucleus index (RD%) across all experimental conditions. Pre‐treatment led to reductions between 71.4% and 81.1%, while simultaneous treatment ranged from 41% to 67%. Post‐treatment with the three highest concentrations of ethanolic extract of açai seed (EEAS) produced reductions of 71%, 67%, and 73%, respectively (see Table 4). These findings were based on cells exposed to cytogenetic damage caused by paracetamol (0.3 mg/mL), which serves as a standard model for oxidative stress (Figure 4). Statistical analysis using ANOVA, followed by Tukey's test (*p* < 0.05), confirmed that the differences between the treated groups and the positive control were significant.

**TABLE 4:** Effect of EEAS on meristematic cells of *Allium cepa*, counting 4000 cells per treatment.

Treatments	Mitotic index (%)	Number of chromosomal aberrations				Apoptosis	Necrosis	Harm reduction (HR%)
		Micronucleus	Anaphasic bridge	C—Metaphase	Bud			
NC	2.25 ± 0.31 a	0.4 ± 0.54 a	0.00	0.00	0.00	0.4 ± 0.54 a	0.00	—
PC	0.7 ± 0.20 b	10.2 ± 1.3 b	2.8 ± 0.83 b	2 ± 1 b	2.4 ± 1.51 b	7.6 ± 1.14 b	16.6 ± 2.7 b	—

Pre‐treatment
25	0.92 ± 0.18 b	3.2 ± 0.83 c	0.00	0.00	0.8 ± 0.8 c	3.4 ± 1.14 c	7.8 ± 1.3 c	77.99
50	1.22 ± 0.25 b	2.2 ± 1.30 a	0.2 ± 0.44 a	0.00	0.4 ± 0.54 c	2.8 ± 0.83 c	4 ± 1 c	93.08
100	1.22 ± 0.34 b	2.2 ± 0.83 a	0.6 ± 0.89 a	0.00	0.4 ± 0.54 c	3.6 ± 1.67 c	2.6 ± 1.14 a	88.05
200	1.43 ± 0.22 c	2.25 ± 1.14 a	0.5 ± 0.54 a	0.00	0.5 ± 0.54 c	2.7 ± 0.83 c	3 ± 1.3 a	87.42

Simultaneous treatment
25	1.67 ± 0.18 b	5.4 ± 1.51 c	0.00	0.00	0.8 ± 0.83 a	6.2 ± 1.48 b	12.6 ± 4.66 b	37.74
50	1.92 ± 0.22 c	5.6 ± 1.51 c	0.00	0.00	0.8 ± 0.83 a	6.8 ± 1.78 b	11.2 ± 3.11 c	25.16
100	2.67 ± 0.66 a	4.4 ± 1.1 c	0.00	0.00	0.6 ± 0.54 a	2.6 ± 1.14 a	9.6 ± 1.5 c	47.80
200	2.72 ± 0.28 a	4.4 ± 1.14 c	0.00	0.00	0.6 ± 0.5 a	3.2 ± 1.48 c	3.6 ± 1.81 a	67.30

Post‐treatment
25	1.47 ± 0.44 b	5.8 ± 0.83 c	0.00	0.00	0.2 ± 0.44 a	3.4 ± 1.14 c	7.4 ± 1.14 c	52.83
50	1.45 ± 0.32 b	3.2 ± 0.83 c	0.2 ± 0.44 a	0.2 ± 0.44 a	0.00	3.4 ± 1.14 c	5.2 ± 1.3 c	83.02
100	1.69 ± 0.31 a	3.6 ± 1.14 c	0.00	0.4 ± 0.89 a	0.00	4.8 ± 1.92 c	4.4 ± 1.14 c	67.34
	1.86 ± 0.53 a	3 ± 0.70 c	0.00	0.00	0.00	4.4 ± 1.14 c	2.2 ± 0.83 a	73.46

*Note*: Data expressed as mean ± standard deviation; same letters represent (“a” and “b”) treatments with no significant difference (*p* > 0.05) and “c” represents different values of NC and PC.

**FIGURE 4:**
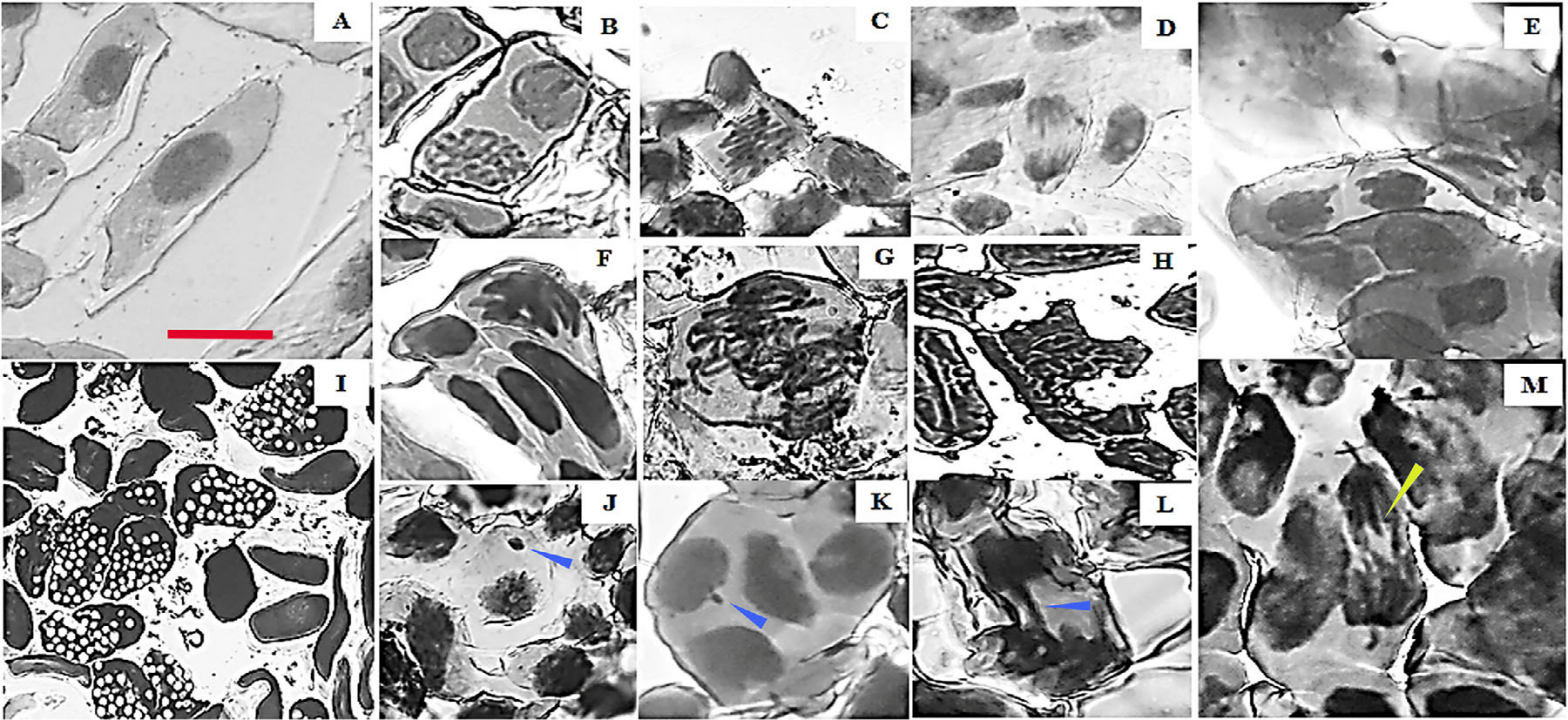
Micrographs of *Allium cepa* cells at different stages of the cell cycle, observed in root preparations. (A) interphase; (B) prophase; (C) metaphase; (D) anaphase; (E) telophase; (F) C‐metaphase; (G) polyploidy; (H) necrosis; (I) apoptosis; (J) micronucleus (blue arrow head); (K) nuclear bud (blue arrow head); (L) anaphase bridge (blue arrow head); (M) anaphase bridge with fragment (yelow arrow head). Red bar = 10 µm. *Source*: authors.

### 3.7 Clonogenic Assay

The MNP01 cell line exhibited a significant difference between treatments only at the 200 µg/mL concentration of extract, with no notable change in colony formation (Figure 5). Although the results were not statistically different from each other, there was a numerical decrease toward decreased average colony formation as extract concentrations increased. In the ACP02 cell line, extract treatments did not show any significant differences compared to each other or to DMSO, except at 200 µg/mL and with PC. A reduction in average colony formation was also noted with higher extract concentrations, though these changes were not statistically significant. For both cell lines, the cytoprotection assay showed no colony formation and no statistically significant difference in PC (Figure 6).

**FIGURE 5:**
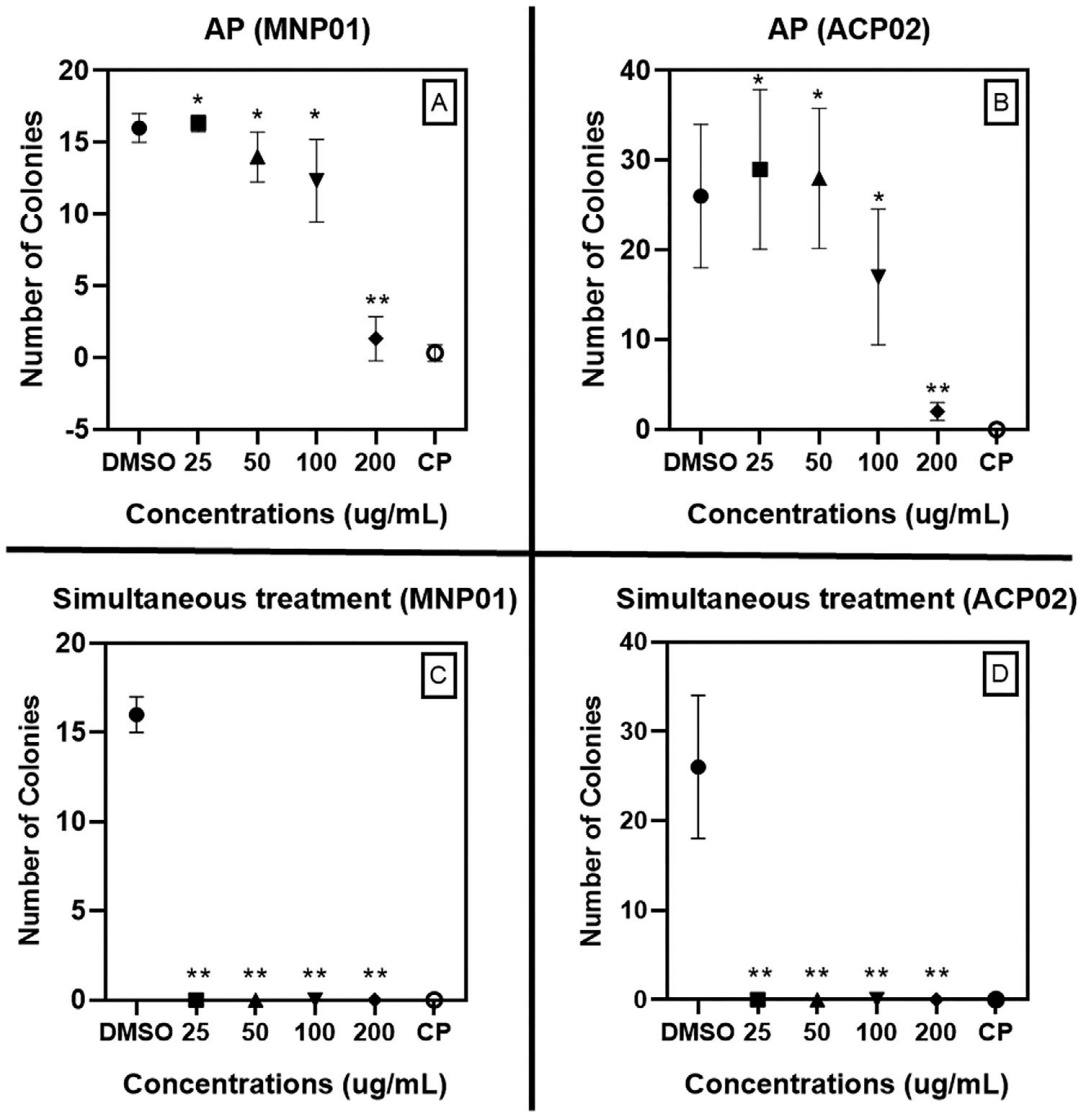
Strains subjected to the colony formation assay. (A) MNP01 exposed to different concentrations of the extract (25–200); (B) ACP02 exposed to different concentrations of the extract (25–200); (C) MNP01 exposed to the extract + Cisplatin; (D) ACP02 exposed to the extract + Cisplatin. Values expressed as number of colonies formed ± standard deviation. *indicates statistical similarity between treatments and with DMSO; ** indicate statistical similarity with PC.

**FIGURE 6:**
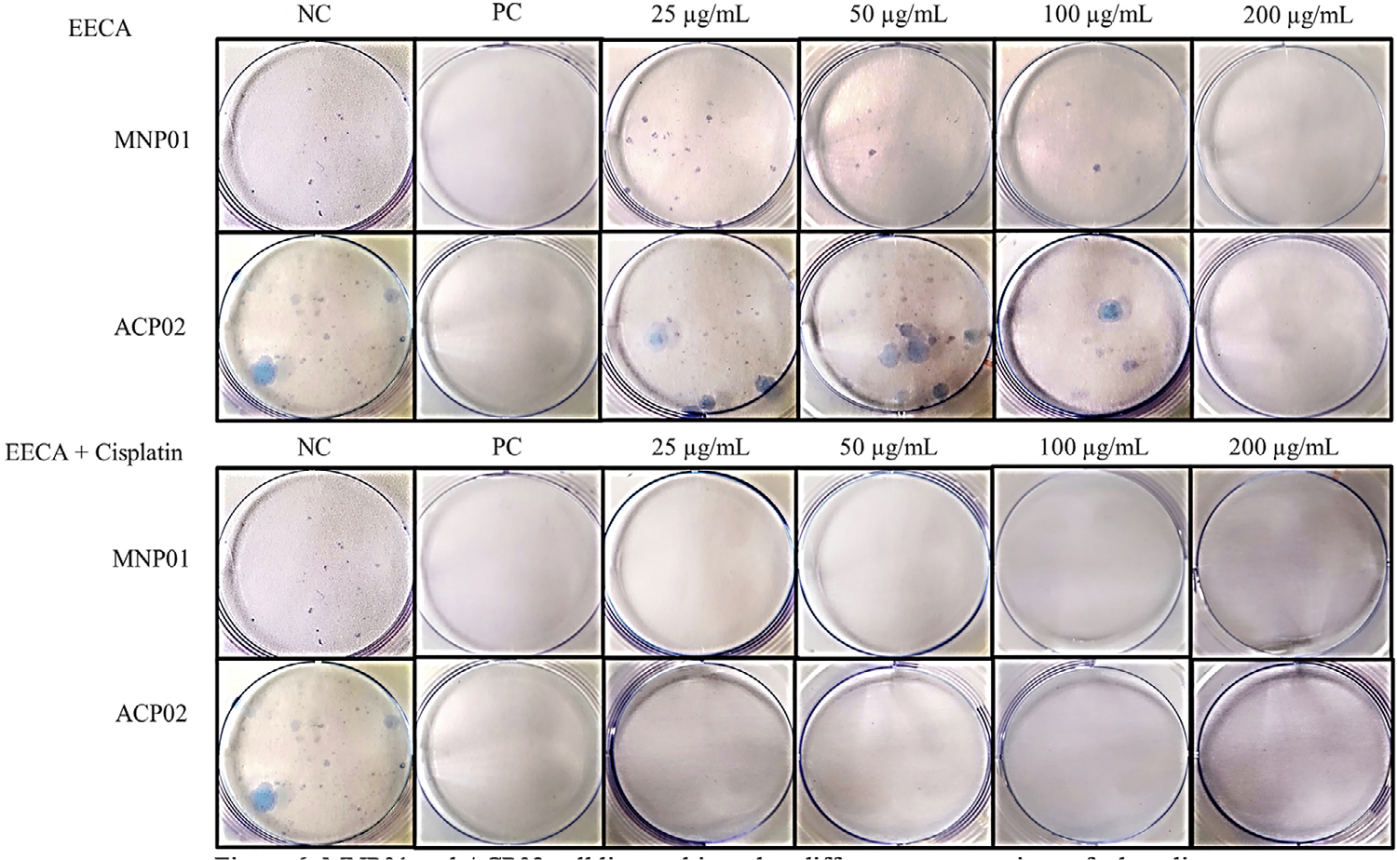
MNP01 and ACP02 cell lines subjected to different concentrations of ethanolic extract of açaí seed (EEAS) in the colony formation assay with and without the presence of cisplatin (20 µM). As the intrinsic toxicity and cytoprotection assays were performed simultaneously using the same experimental setup, they share the same representative control group. Consequently, the control group is shown twice, as it serves as the control for both assays. NC = negative control; PC = positive control. *Source*: author.

## 4 Discussion

Minor quantitative variations in the chemical compositions of BA and WA extracts may account for the observed differences in antioxidant performance, as BA demonstrates 30% greater efficacy in neutralizing 50% of DPPH free radicals compared to WA. This distinction could be attributed to the presence of benzoic acid in BA—which is absent in WA—as benzoic acid functions as a natural preservative [36]. Its inclusion in the extract may enhance the activity of other compounds, thereby contributing to the superior antioxidant potential of BA.

In the MTT cytotoxicity assay, cell viability decreased by no more than 20% at the tested concentrations, precluding calculation of the IC50 value. Additionally, the micronucleus test demonstrated no evidence of genotoxic activity. Notably, wet analysis identified the presence of condensed tannins—also referred to as proanthocyanidins or procyanidins—in both BA and WA extracts as a phytochemical constituent. Although this class of compounds has been previously reported to possess toxic potential, including in in vitro studies with cancer cell lines [37, 38], their apparent lack of cytotoxicity here suggests they are present only in small amounts or may exert antagonistic interactions with other compounds that mitigate their toxicity. Such interactions, where combinations of constituents diminish toxicity and adverse effects, are well documented in herbal preparations and can enhance the therapeutic efficacy of medicinal plants [39].

The differential sensitivity observed between normal gastric epithelial (MNP01) and gastric adenocarcinoma (ACP02) cells—where normal cells were affected at 400 µg/mL while cancer cells remained viable—warrants careful consideration considering the existing literature on açaí seed extracts. Published studies consistently report that açaí seed extracts exhibit selective cytotoxicity toward various cancer cell lines, including breast (MCF‐7), prostate (DU145 and LNCaP), lung (A549 and NCI‐H460), liver (HepG2), and cervical (HeLa) cancer cells, while showing no toxicity to non‐tumor cells [40, 41]. This selectivity has been attributed to the rich phenolic composition of açaí seeds—particularly proanthocyanidins and catechins—which can induce reactive oxygen species (ROS) production and trigger cell death pathways preferentially in malignant cells. This highlights a crucial aspect of açaí seed extracts: they exhibit biphasic effects—cytoprotective at lower concentrations through antioxidant mechanisms, and potentially cytotoxic at higher concentrations when antioxidant defenses are overwhelmed. Our results underscore that the cytotoxic response to açaí seed extracts is cell line‐dependent and context‐specific. The lack of effect on ACP02 cells at the concentrations tested does not negate the potential anticancer properties of açaí seeds but rather suggests that gastric adenocarcinoma cells may respond differently than breast, cervical, or prostate cancer cells. Future studies investigating the specific molecular pathways—including p53 status, Nrf2‐mediated antioxidant responses, and drug efflux mechanisms—in these cell lines would be valuable to elucidate the mechanisms underlying this differential sensitivity.

The cytoprotective activity observed in the extracts is likely attributable to their substantial antioxidant capacity, which is primarily associated with phenolic compounds present in the seed extracts. Notable constituents include catechollate, catechin, and 3,4‐dihydroxybenzoic acid—examples of phenols that may contribute to scavenging excess free radicals generated by DOX. Previous studies have demonstrated that DOX exhibits pro‐oxidant activity during its metabolic activation and may also diminish the organism's antioxidant defense mechanisms, leading to various toxic side effects during cancer treatment [42, 43].

In the *A. cepa* assay, the significant decrease in micronucleus formation during pre‐treatment (71.4%–81.1%) suggests that BA may exert a protective, antimutagenic effect. Although the underlying mechanisms were not directly investigated in the present study, one possible explanation is the prevention of ROS‐induced DNA damage. It is tempting to speculate that this effect could be related to the activation of the Nrf2/Keap1 pathway, a master regulator of antioxidant gene expression [44]. Similarly, protection against paracetamol‐induced hepatotoxicity has been attributed to the modulation of enzymatic defense systems [45]. Taken together, these findings from the literature raise the hypothesis that BA pre‐treatment may act by directly neutralizing ROS and/or modulating cellular defense pathways. However, further studies—including molecular assays such as qPCR or Western blotting—are necessary to confirm the involvement of the Nrf2/Keap1 pathway in the antimutagenic activity of BA observed in our model.

During the post‐treatment phase, the observed reduction in the micronucleus index (71%–73%) suggests a bioantimutagenic effect, which may be attributable to the possible stimulation of DNA repair mechanisms following paracetamol exposure [46]. Although this hypothesis was not directly tested in our experimental model, previous studies provide a plausible mechanistic framework. For instance, it was demonstrated that Nrf2 activation can enhance the expression of 8‐oxoguanine DNA glycosylase (OGG1), a key enzyme involved in the excision of 8‐oxo‐dG, a marker of oxidative DNA damage [47]. In a similar speculative vein, phloroglucinol has been reported to activate extracellular signaling pathways, such as ERK kinases, which may facilitate cell proliferation and survival under stress conditions [48]. It is therefore conceivable that EEAS could exert a dual effect—providing antioxidant protection while also supporting tissue regeneration—although this remains a hypothesis to be tested in future investigations. Molecular studies, including the evaluation of OGG1 expression and ERK phosphorylation, are warranted to confirm the mechanisms underlying the bioantimutagenic activity of EEAS observed in the post‐treatment phase.

Simultaneous treatment shows moderate effectiveness (41%–67%), indicating a complex interaction between ACE inhibitors and paracetamol. Paracetamol is broken down by CYP2E1 and CYP3A4 enzymes, which produce N‐acetyl‐p‐benzoquinone imine (NAPQI), a harmful byproduct that depletes glutathione and causes oxidative stress [49]. It was found that plant phenols can influence these cytochromes and affect how quickly NAPQI forms [50]; also, phloroglucinol may interfere with CYP3A4 and compete with other drugs [51]. Still, if concentrations are too low, this inhibition might not stop NAPQI from forming or effectively compete with antioxidant enzymes, which could explain why efficacy is reduced at lower levels [52].

The combined effects of compounds found in EEAS may help explain the results seen in this experiment. For example, while phloroglucinol serves as an antioxidant and modulates Nrf2 directly, other flavonoids could enhance its activity by helping regenerate the body's own antioxidants [53]. Such synergy may account for EEAS's effectiveness in both pre‐ and post‐treatment contexts. Furthermore, EEAS's capacity to protect cells at different stages highlights its promise for treating conditions linked to oxidative stress, like drug‐induced liver toxicity [54]. Still, additional in vitro and in vivo pharmacokinetic studies are necessary to determine how clinically useful the extract is, considering factors such as bioavailability, synergy, and safety in animal models.

The clonogenic assay showed that EEAS did not significantly decrease colony formation in either the normal cell line (MNP01) or the cancer cell line (ACP02) at concentrations ranging from 25–100 µg/mL. Only at 200 µg/mL was a reduction observed, which supports the extract's safety at therapeutic levels. The main chemicals in the extract—phloroglucinol (24.71%), catechol‐lactate (18.79%), and 3,4‐dihydroxybenzoic acid (15.12%)—contribute to this effect, as phenolic compounds can act as redox modulators at low doses, helping neutralize reactive oxygen species (ROS) without affecting cell growth [55].

The concentration of 200 µg/mL, which exhibited a statistically significant effect in both cell lines, serves as a critical safety threshold; concentrations exceeding this level may diminish the extract's protective properties and induce cytotoxic or cytostatic actions. Notably, fatty acids constitute a substantial proportion of the extract (36.27%), with palmitic acid (12.11%) and myristic acid (9.35%) being predominant—both compounds are traditionally implicated in inflammatory responses at defined concentrations. These fatty acids can influence cellular membrane rigidity and elicit adaptive stress mechanisms in normal cells [56]. Importantly, non‐toxic levels of palmitic acid stimulate the synthesis of molecular chaperones such as HSP70, which defend proteins against oxidative denaturation—a process potentially underlying the cytoprotective effects observed in the *A. cepa* assay.

A slight decrease was seen as concentrations increased, although this was not statistically significant and probably shows the sublethal effects of phenolic compounds over time. 3,4‐Dihydroxybenzoic acid is known for inducing apoptosis and autophagy in cancer cells, as well as providing protection and antioxidant benefits to normal cells [57]. As these results are not statistically significant, we mention it as exploratory and hypothesis‐generating, since the lack of statistical significance prevents us from drawing definitive conclusions.

However, these effects become statistically meaningful only at higher concentrations (200 µg/mL), since they rely on inhibiting multiple pathways at once to bypass cell repair mechanisms. Similarly, research with phloroglucinol has shown it can suppress gastric cancer cell growth by affecting redox balance, which may result in G1 phase cell cycle arrest [58]. These findings are significant because they demonstrate that the extract does not shield cancer cells from chemotherapy but could offer short‐term protection to non‐cancerous cells.

Short‐term assays like the MTT assay evaluate cell viability and metabolic activity, while clonogenic assays measure long‐term genomic stability and DNA repair capacity [59]. The extract may preserve short‐term cell survival but fail to prevent cisplatin‐induced irreversible DNA damage, despite containing compounds such as phloroglucinol known to stimulate DNA repair [47, 58]. This highlights the importance of using both short‐ and long‐term assays to accurately assess cytoprotective agents. Future research should validate these findings in complex models, including normal/cancerous cell co‐cultures, migration assays, and endogenous antioxidant biomarkers, to better simulate clinical scenarios and investigate the synergy of BA components in genomic protection.

Combining this chemotherapeutic agent with drugs that have strong antioxidant properties, including those derived from açai, may help reduce short‐term treatment effects. This finding is valuable, as açai seeds—typically agro‐industrial waste—could be repurposed for various products. Previous studies have highlighted the high antioxidant potential of fruit seeds that are often discarded [60, 61, 62, 63].

We recommend that future research should assess how these extracts protect cells from other forms of toxicity, including genotoxicity and mutagenicity, in both cancerous and non‐cancerous cell lines.

Additionally, it is important to analyze the optimal timing for administering the protective agent—whether before, during, or after exposure to the harmful substance. Determining the most effective concentrations of these mixtures is essential for fighting cancer while protecting healthy cells, especially since cancer cells tend to be more resistant. Cancer cells have unique features, such as their ability to evade cell death and changes in mitochondrial numbers, which complicate the task of identifying the best exposure method and concentration with minimal toxicity [64, 65].

## 5 Conclusion

Our findings indicate that both white and black açai seed extracts demonstrate strong antioxidant properties, with the black açai extract showing slightly greater effectiveness than the white. Nonetheless, we recommend further research into the biological activities of both, including their potential to protect organisms from damage caused by daily exposure to external factors that promote excessive free radical formation. The ability to capture these molecules is linked to preventing or reducing symptoms of various diseases, such as cancer, diabetes, and cardiovascular conditions.

We further noted the absence of cytotoxic and genotoxic effects at the tested conditions and concentrations. The observed cytoprotective activity proved highly relevant to this work, informing subsequent steps undertaken by our research group. Additionally, these findings provide valuable information for other researchers assessing extracts derived not only from açai fruit pulp but also from its seed.

## Author Contributions

L.F.C.A. and E.T.P.C. contributed to the conceptualization, methodology, investigation, and writing of the original draft. H.C.G.S. and O.V.M.S. were responsible for formal analysis, investigation and data curation, writing, review, and editing. G.H.S.L., K.M.M., V.S.C., and A.O.A.V. were responsible for investigation, methodology, data curation, writing, review, and editing. J.D.R. and K.A.D.C. were responsible for methodology, formal analysis, validation and writing, review, and editing. A.S.S. contributed to investigation, formal analysis, resources, visualization and writing, review, and editing. C.Y.N. and J.C.P. provided supervision, funding acquisition, project administration, resources and contributed to the writing, review, and editing of the manuscript. All authors have read and agreed to the published version of the manuscript.

## Funding

This research was funded by the Coordination for the Improvement of Higher Education Personnel (CAPES) to C.Y.N (047/2012) and by the National Bank for Economic and Social Development to J.C.P (2.318.697.0001). C.Y.N (307170/2021‐7) and JCP (307154/2021‐1) would like to thank CNPq for the productivity grants.

## Conflicts of Interest

The authors declare no conflicts of interest.

## Supporting information




**Supporting File**: cbdv71858‐sup‐0001‐SuppMat.pdf.

## Data Availability

The data supporting the conclusions of this study are described and included in this manuscript and in the supplementary files.
