# Supplementary material for: Comparative Evaluation of Ethanolic Extracts Obtained From the Seeds of White and Black Açai Exhibit Antioxidant and Cytoprotective Activity In Vitro
Source: Chem Biodivers. 2026 Oct 22;23(10):e71858. doi: 10.1002/cbdv.71858 (PMC13691405; doi:10.1002/cbdv.71858)
Supplement: Supplementary file 1 [file CBDV-23-e71858-s001.pdf]

Figure S1: White açai original GC/MS chromatogram.

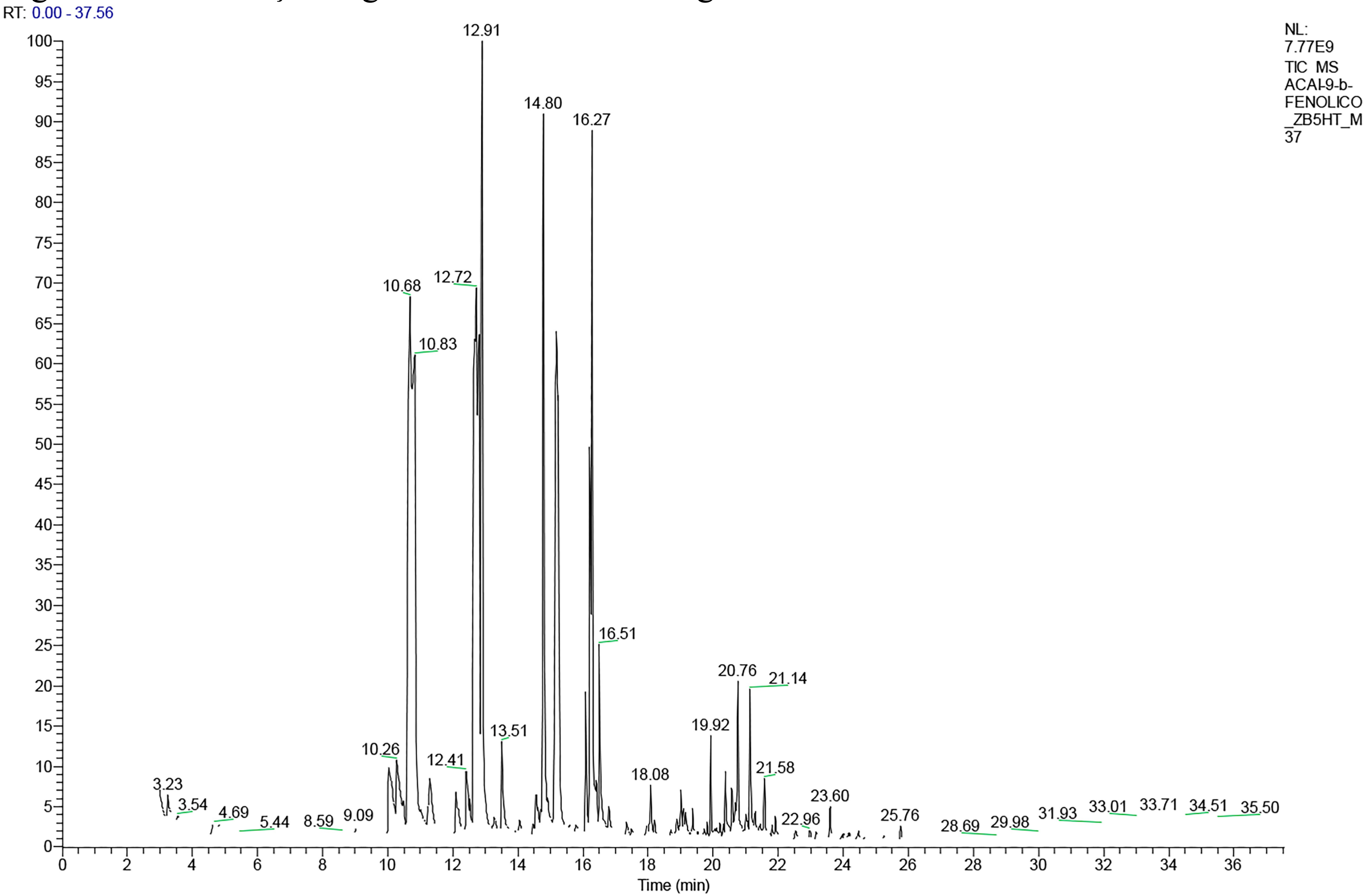

Figure S2: Black açai original GC/MS chromatogram.

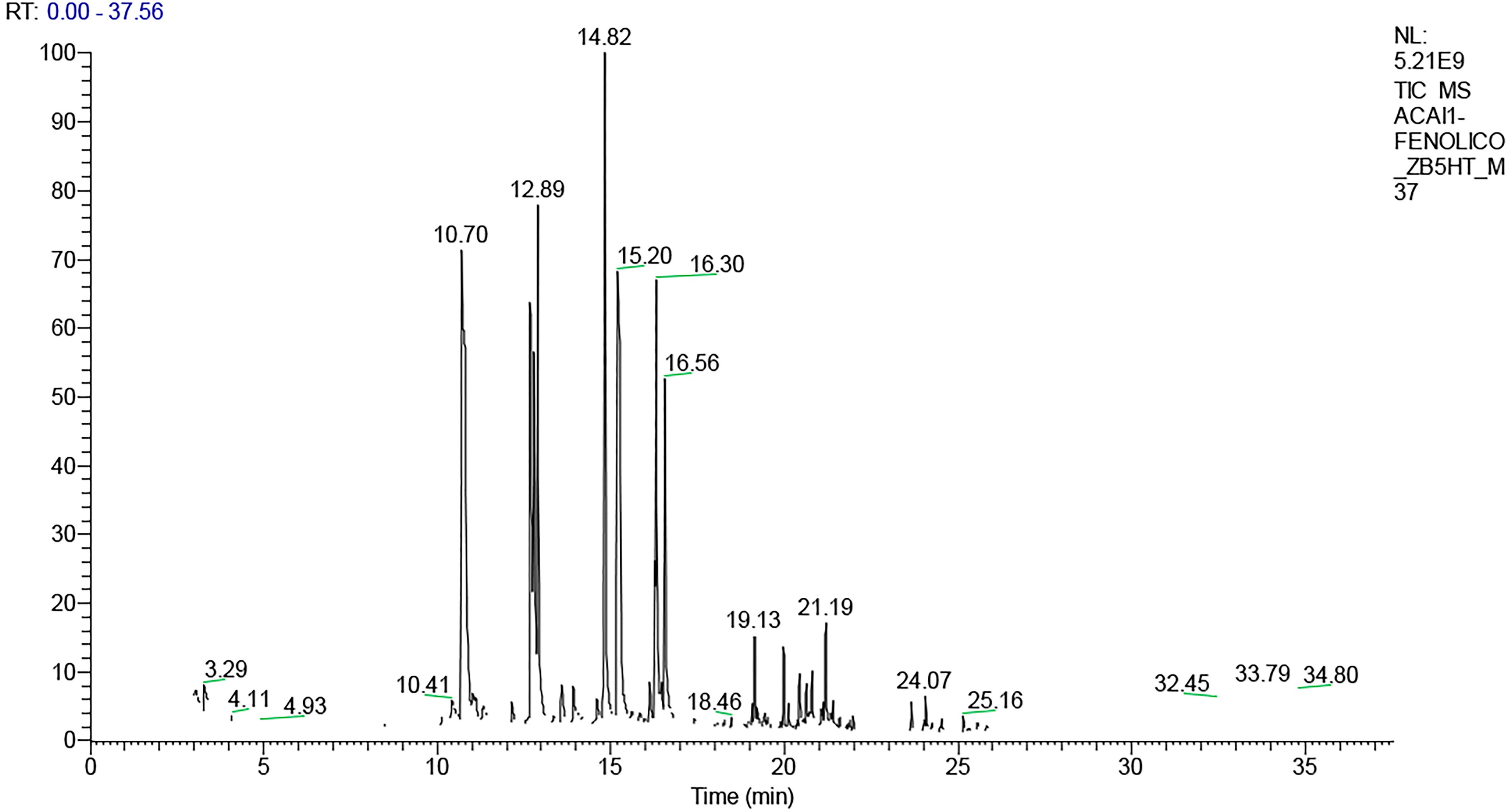

Table S1: Black Açai. Data represent mean of three independent experiments (n=3), extracted from the dose-response curve shown in Figure 1.

Coefficient of Determination (R^2): 0.9385

Half maximal effective concentration (EC_{50}): 9.8 µg/mL Table of Experimental Data

Concentration (µg/mL)	DPPH capture (%)
2	~ 48%
3	~ 51%
4	~ 57%
5	~ 71%
6	~ 91%
7	~ 96%
8	~ 97%

Footnote: DPPH capture percentage values were estimated by graphical approximation based on the scattered points plotted in the corresponding original graphs.

Table S2: White Açai. Data represent mean of three independent experiments (n=3), extracted from the dose-response curve shown in Figure 1, Coefficient of Determination (R^2): 0.9201

Half maximal effective concentration (EC_{50}): 14.47 µg/mL Table of Experimental Data

Concentration (µg/mL)	DPPH capture (%)
2	~ 44%
3	~ 46%
4	~ 51%
5	~ 64%
6	~ 89%
7	~ 99%
8	~ 97%

Footnote: DPPH capture percentage values were estimated by graphical approximation based on the scattered points plotted in the corresponding original graphs.
